# Non-thrombotic pulmonary emboli: imaging findings and differential diagnoses

**DOI:** 10.1007/s11604-025-01829-y

**Published:** 2025-07-02

**Authors:** Nanae Tsuchiya, Yuma Chinen, Kazuyoshi Yumoto, Masaki Uechi, Junji Ito, Kimei Azama, Wataru Makino, Tomomi Koga, Akihiro Nishie

**Affiliations:** 1 Department of Radiology, Graduate School of Medical Science, University of the Ryukyus, 1076 Kiyuna, Ginowan-Shi, Okinawa 901-2720 Japan; 2 Department of Radiology, Nakagami Hospital, Okinawa, Japan

**Keywords:** Nonthrombotic pulmonary embolism, Fat embolism, Tumor embolism, Air embolism, CT, Pulmonary infarction

## Abstract

**Objective:**

Nonthrombotic pulmonary embolism (NTPE) encompasses a diverse group of rare but clinically significant conditions that involve fat, tumor, air, amniotic fluid, septic, parasitic, and iatrogenic emboli. This review summarizes the pathophysiology, imaging findings, and differential diagnoses of NTPE toward the goal of facilitating its accurate radiologic diagnosis.

**Content:**

In NTPE, the use of computed tomography pulmonary angiography (CTPA) may reveal filling defects in the pulmonary arteries; however, the embolus is often not directly visualized, especially in cases of microscopic emboli. NTPE-related pulmonary lesions arise from two mechanisms: mechanical obstruction and chemical injury. Clarifying the link between these mechanisms and imaging features is crucial for achieving the accurate diagnosis of NTPEs. Tools such as dual-energy CT and ventilation-perfusion scintigraphy contribute to the detection of microvascular involvement. We present the characteristic imaging patterns and diagnostic pitfalls for each NTPE subtype, illustrated with representative cases.

**Conclusion:**

The awareness of NTPE entities and their imaging features is essential for the timely diagnosis and management of these emboli. Radiologists should consider the possible presence of an NTPE in patients with unexplained respiratory symptoms, especially when conventional imaging fails to demonstrate thrombotic emboli.

## Introduction

A pulmonary embolism is a potentially life-threatening condition characterized by the occlusion of pulmonary arteries. The most prevalent form of a pulmonary embolism is a thrombotic embolism, but nonthrombotic emboli are also observed; the term ‘nonthrombotic pulmonary embolism (NTPE)’ encompasses a heterogeneous group of rare but clinically significant entities that include embolization by fat, tumor cells, gas, amniotic fluid, septic material, parasitic organisms, and foreign bodies [1–3]. The presence of an NTPE poses a formidable diagnostic challenge due to the diverse etiologies and often nonspecific clinical presentations such as acute respiratory distress, hypoxia, and hemodynamic collapse. Unlike thrombotic emboli, nonthrombotic emboli are frequently invisible on computed tomography pulmonary angiography (CTPA), necessitating radiologists to recognize indirect findings such as mosaic perfusion and signs of right ventricular overload [4–6]

This review provides a comprehensive overview of NPTEs from a radiologic perspective, highlighting key imaging features, the underlying pathophysiology, and practical diagnostic tips. By integrating representative case examples and summarizing the relevant literature, we hope to enhance the diagnostic accuracy and awareness of NPTEs among radiologists and clinicians who treat patients with pulmonary vascular diseases.

## NTPE pathophysiology and imaging modalities

### Pathophysiology and imaging differences on chest CT: Mechanical and chemical mechanisms

An NTPE can present with a variety of imaging findings depending on the size and nature of the embolus. Macroscopic emboli may obstruct the main, lobar, or segmental pulmonary arteries, with CTPA typically demonstrating filling defects in the affected vessels [2, 7–9]. This finding is considered directly diagnostic. Microscopic emboli are not visible on CTPA, but CTPA may reveal the following: mosaic attenuation or pulmonary infarction suggestive of a peripheral pulmonary artery embolism, discrepancies in the diameters of the pulmonary arteries and veins, and signs of right heart strain [1, 7–9]. It is important that radiologists and clinicians be aware of the possibility of the presence of an NTPE based on these indirect findings.

In addition, variations in the characteristics of emboli influence their chemical reactivity, resulting in lung abnormalities due to differing degrees of chemical injury and the inflammatory response [9–11]. Pulmonary lesions caused by an NTPE are sometimes nonspecific and complex, but the relationship between the underlying pathology and the imaging findings can be more easily understood if emboli are categorized into lesions resulting from mechanical obstruction and those caused by chemical reactions (Table 1).

**Table 1:** Imaging features of NTPE based on the underlying pathology

Mechanism	Mechanical	Chemical
*Direct findings*	Filling defect in PA	None
*Indirect findings:*		
Cardiovascular	・Right ventricular and pulmonary artery dilation ・Collapse of the pulmonary veins and left atrium ・Ventricular septal deviation ・Reflux of contrast medium into the IVC and hepatic veins	
Lung parenchyma	・Small centrilobular nodules ・Geographic ground-glass opacities (mosaic perfusion) ・Hydrostatic pulmonary edema ・Pulmonary infarction	・Patchy ground-glass opacity ・Crazy-paving pattern ・Airspace consolidation

Mechanically induced emboli can produce central filling defects or arterial occlusions that are visible on CTPA. Examples include fat globules (− 30 Hounsfield units [HU] or less), tumor fragments (enhanced lesion), or foreign bodies (i.e., high-density material) [2, 4, 10]. The pulmonary lesions that are due to mechanical obstruction include neovascularization, mosaic perfusion, hydrostatic pulmonary edema, and pulmonary infarction. Small (< 1 cm), ill-defined centrilobular and subpleural nodules are sometimes seen on CT during the acute phase of fat embolism syndrome (FES). These nodules are presumed to represent microhemorrhage and neovascularization following mechanical obstruction or ischemia [1, 12, 13].

A geographic distribution of ground-glass opacities (GGOs) within secondary lobular units has also been reported, attributed to variations in pulmonary perfusion (mosaic perfusion) [4]Areas of high perfusion exhibit ground-glass opacity, while embolized, low-perfusion areas remain preserved, thus producing a mosaic perfusion pattern. Distinguishing mosaic perfusion from small airway disease relies on the evaluation of the affected pulmonary artery’s diameter, as this diameter is typically enlarged in embolic disease but normal in airway pathology [4]. The presence of a peripheral pulmonary artery embolism reduces the pulmonary capillary bed, leading to pulmonary hypertension[14]. The consequent relative increase in the blood flow to unaffected pulmonary arteries raises the hydrostatic pressure in those areas, causing hydrostatic pulmonary edema [14].

The typical CT findings of pulmonary infarction include wedge-shaped, pleural-based opacity with bulging convex borders and linear opacities extending toward the hilum [15]. A pulmonary infarction may present as central lucency within areas of peripheral consolidation (indicated by the reversed halo sign or atoll sign) or as regions of reduced enhancement, indicating necrosis or ischemia with secondary cavitation [15].

Another mechanism underlying abnormal findings in the lung fields is a chemical injury. Chemical reactions triggered by an embolus damage vascular endothelial cells, leading to increased vascular permeability and pulmonary edema. This process underlies the pathology of acute respiratory distress syndrome (ARDS) [2]. The common CT findings in ARDS caused by an NTPE are patchy GGOs, often accompanied by interlobular septal thickening, which may create a ‘crazy-paving’ pattern. In severe cases, consolidation appears, indicating more extensive pulmonary hemorrhage and edema [2, 4, 11, 14, 16].

### Imaging modalities

CTPA cannot diagnose all cases of NTPE; however, it should be performed as the first-line examination. The standard imaging protocol for CTPA is shown in Table 2 [17]. CT findings and typical pitfalls across subtypes of NTPE are summarized in Table 3.

**Table 2:** The standard imaging protocol for CTPA

Scanner	Multidetector CT (64-slice or higher recommended); ECG-gating not required
Patient position	Supine position; arms raised above the head
Scan range	From lung apex to below the diaphragm (to include peripheral pulmonary arteries)
Scan mode	Helical scan with low pitch and high scan speed
Scan timing	Bolus tracking technique: ROI on main pulmonary artery (threshold: 100–150 HU); scan starts 3–5 s after threshold is reached Fixed delay technique: PA 15–25 s (adjusted by injection rate), venography 210 s
Contrast medium	Iodinated contrast: PA 400 mgI/kg, PA + Venography 600 mgI/kg) Injection rate: 3–5 mL/sec Followed by 20–40 mL saline flush
Image reconstruction	Slice thickness: 0.5–1.0 mm Window width/level: W700/L100 (vessels), W1500/L–600 (lung parenchyma) MPR, MIP, CPR, and VR reconstructions are useful

*CT* computed tomography, *ECG* electrocardiogram, *ROI* region of interest, *PA* pulmonary artery, *MPR* multiplanar reconstruction, *MIP* Maximum intensity projection, *CPR* curved planar reformation, *VR* volume rendering

**Table 3:** CT findings and typical pitfalls of NTPEs

NTPE subtype	CT findings	Typical pitfalls
Amniotic fluid embolism	Diffuse bilateral GGOs, septal thickening, consolidations (lower lobes), right heart strain, absence of filling defects	Nonspecific findings; pregnant women are at risk of thromboembolism
Fat embolism	Bilateral GGOs, interlobular septal thickening, patchy consolidations, small centrilobular nodules,'crazy-paving'pattern, fat-density emboli (rare)	Mimics ARDS or contusion; overlooked in absence of trauma
Tumor embolism/PTTM	Macro: filling defects with enhancement, filling defects persist despite anticoagulation Micro: multifocal infarcts, centrilobular nodules, tree-in-bud, septal thickening PTTM: signs of pulmonary hypertension	Mimics thromboembolism or CTEPH; FDG-PET is useful for the diagnosis
Septic embolism	Peripheral cavitary nodules, wedge-shaped opacities, feeding vessel sign, halo sign, pleural effusion	Confused with malignancy or thromboembolism; cavitation and systemic signs of infection are clues
Parasitic embolism	Dirofilariasis: well-defined peripheral nodules Echinococcosis: cystic intra-arterial lesions Schistosomiasis: enlarged PA, mosaic perfusion	Mimics malignancy or granulomatous disease; often misdiagnosed outside endemic areas
Iatrogenic embolism	High-density foreign bodies in PA (linear or punctate), tubular hyperdense opacities, use bone window for metallic objects	Mistaken for thromboembolism or calcified emboli; differentiate from Intravascular devices placed for therapeutic purposes
Gas embolism	Low-attenuation gas in PA, RV, or systemic arteries; pulmonary hypertension in decompression illness	Gas may vanish before imaging examination; misdiagnosed as minor or irrelevant

*NTPE* non-thrombotic pulmonary emboli, *CT* computed tomography, *GGO* gland glass opacity, *ARDS* acute respiratory distress syndrome, *PTTM* pulmonary tumor thrombotic microangiopathy, *CTPH*: chronic thromboembolic pulmonary hypertension, *FDG-PET* Fluorodeoxyglucose-positron emission tomography, *PA* pulmonary artery, *RV* right ventricular

Ventilation-perfusion (V/Q) scintigraphy remains useful, especially in patients for whom the use of CT is contraindicated or equivocal (e.g., pregnant woman and patients who are allergic to intravenous contrast agents). V/Q scintigraphy can identify perfusion defects in the absence of ventilation abnormalities, but its specificity for NTPEs is lower compared to that for a thrombotic pulmonary embolism (PE) [4].

Dual-energy computed tomography (DECT) enhances diagnostic performance by assessing iodine perfusion maps, which can reveal perfusion defects that are not evident on conventional CT, which is particularly valuable for detecting the microvascular involvement that is observed in patients with pulmonary tumor thrombotic microangiopathy (PTTM) or microscopic tumor emboli [9, 17, 18].

## Amniotic fluid embolism

### Overview and pathogenesis

An amniotic fluid embolism (AFE) is a rare but frequently fatal obstetric emergency, with an estimated incidence of 2–7.7 per 100,000 deliveries [5, 18]. It typically occurs during labor, a cesarean section, or in the immediate postpartum phase. The pathophysiology involves the entry of amniotic fluid and/or fetal debris (including squamous cells and lanugo) into the maternal venous circulation through a uterine vein rupture or placental tear [18, 19]. Two AFE mechanisms have been proposed: mechanical obstruction of pulmonary arterioles, and an anaphylactoid response mediated by vasoactive substances leading to pulmonary vasoconstriction, capillary leak, and systemic inflammation [7, 18]. Risk factors for AFE include advanced maternal age, placenta previa, multiple gestation, and uterine overdistension [18].

### AFE: imaging findings

**Fig. 1:**
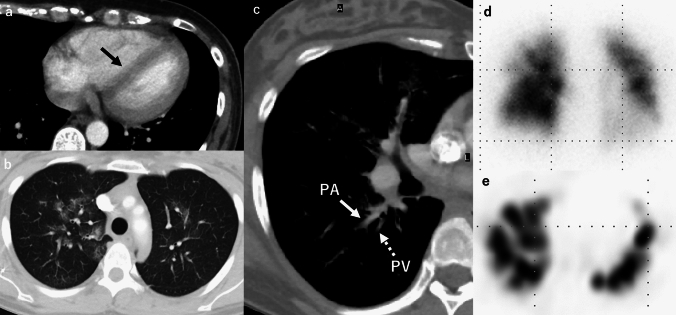
Amniotic fluid embolism (AFE). A woman in her 30 s developed continued bleeding following labor induction and fetal delivery which was controlled by uterine artery embolization (UAE). Persistent hypoxemia was noted after the completion of the UAE. Contrast-enhanced CT revealed right ventricular and pulmonary artery dilation, along with leftward deviation of the interventricular septum (**a**). The lung fields demonstrated multiple GGOs predominantly in the perihilar regions (**b**). The peripheral pulmonary veins (PVs) appeared narrower than the corresponding pulmonary arteries (PAs), suggesting the presence of emboli at the level of the pulmonary capillaries (**c**). 99mTc-MAA lung perfusion scintigraphy revealed multiple fine band-like and wedge-shaped perfusion defects in both lungs, consistent with a peripheral pulmonary arterial embolism (**d**, **e**). Elevated zinc coproporphyrin levels and decreased C1 inhibitor activity were observed, leading to a diagnosis of AFE

The imaging findings for AFE are nonspecific and serve mainly to exclude other diagnoses. Chest radiography often shows bilateral, heterogeneous or homogeneous airspace opacities suggestive of noncardiogenic pulmonary edema or ARDS [4, 18]. On CT, typical AFE findings include diffuse bilateral GGOs, interlobular septal thickening, and consolidations, especially in the lower lobes [5, 7, 20]. Pulmonary arterial filling defects are usually absent. Secondary signs such as right ventricular enlargement (or strain) and pleural effusions may also be present [18, 19]. Figure 1 illustrates a representative AFE case.

### AFE: differential diagnoses and pitfalls

The differential diagnoses for AFE include PTE, sepsis-induced ARDS, diffuse alveolar hemorrhage, and aspiration pneumonia [7, 18]. Pregnant women are at risk of the development of a pulmonary embolism and may develop pulmonary edema due to various perinatal-related causes [18, 21]. Gestational trophoblastic disease may also mimic AFE [5].

Importantly, fetal squamous cells and other debris are occasionally identified in pulmonary vessels during normal deliveries and in patients with unrelated conditions, thus limiting the diagnostic specificity of these findings [1, 18]. A critical diagnostic pitfall is over-reliance on imaging findings; the diagnosis of AFE remains primarily clinical, based on the sudden onset of respiratory and cardiovascular collapse during the peripartum period [18].

## Fat embolism

### Overview and pathogenesis

A fat embolism (FE) occurs when fat globules enter the bloodstream, typically after a long-bone fracture, orthopedic surgery, or trauma. Less common causes include pancreatitis, burns, and liposuction [22, 23]. The classic clinical presentation, FES, comprises a triad: respiratory distress, neurologic dysfunction, and petechial rash [23]. Both mechanical and biochemical theories have been described for the pathogenesis of FEs, as follows. Mechanically, marrow fat enters torn venules, especially after a bone fracture. Biochemically, circulating free fatty acids cause endothelial damage, promoting capillary leakage and inflammation [1, 23]. Histopathologically, FEs appear as vacuolated spaces within pulmonary capillaries or arterioles, with Oil Red O staining confirming the lipid content [22].

### FE: image findings

**Fig. 2:**
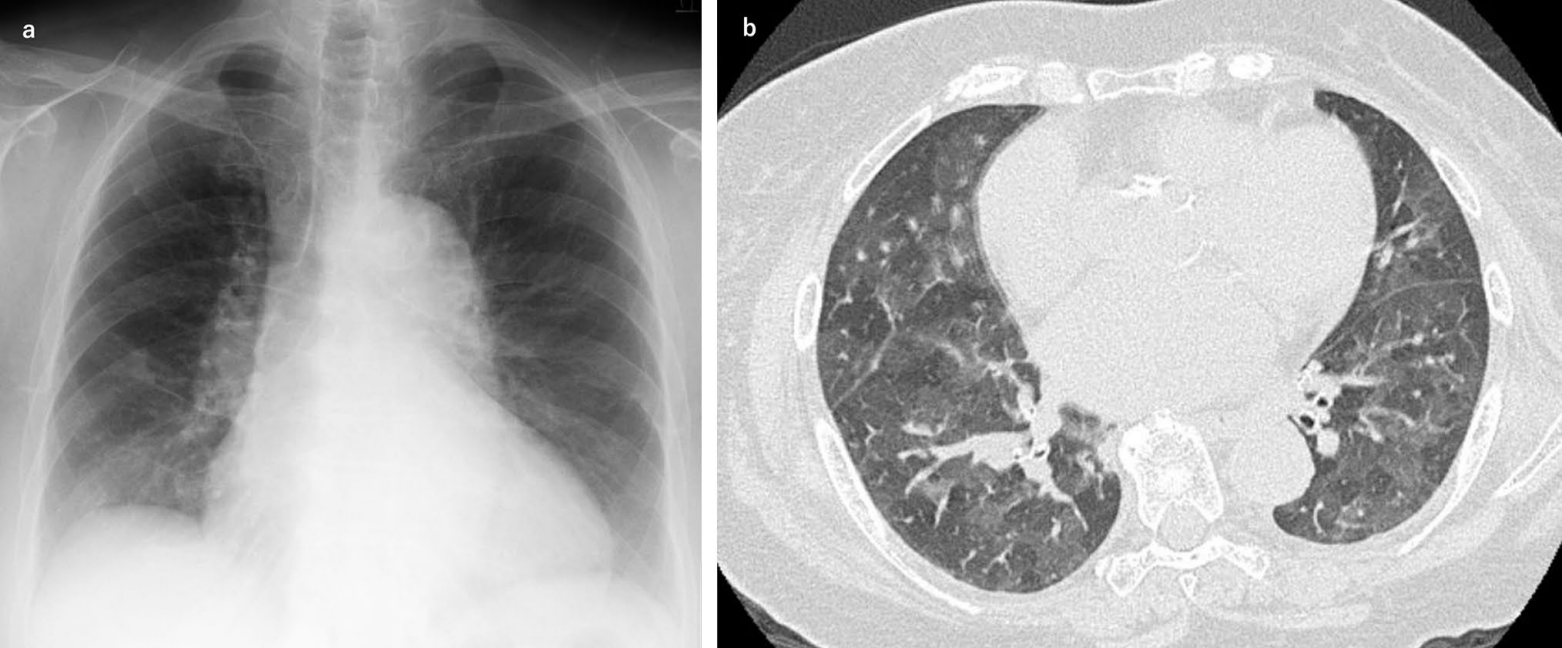
Microscopic pulmonary fat embolism (FE). The patient was a woman in her 90 s who developed acute dyspnea 48 h after experiencing a femoral neck fracture. Chest radiography showed enlarged pulmonary arteries (**a**), and the CT scan revealed patchy GGOs in a mosaic pattern, with the secondary lobule distribution in the segmental or subsegmental territories (**b**)

On chest radiographs, the FE findings are often non-specific but may include diffuse bilateral infiltrates mimicking ARDS [1, 21]. Computed tomography typically reveals bilateral GGOs, interlobular septal thickening, and patchy consolidations [1, 4, 21]. A representative FE case is depicted in Fig. 2.

**Fig. 3:**
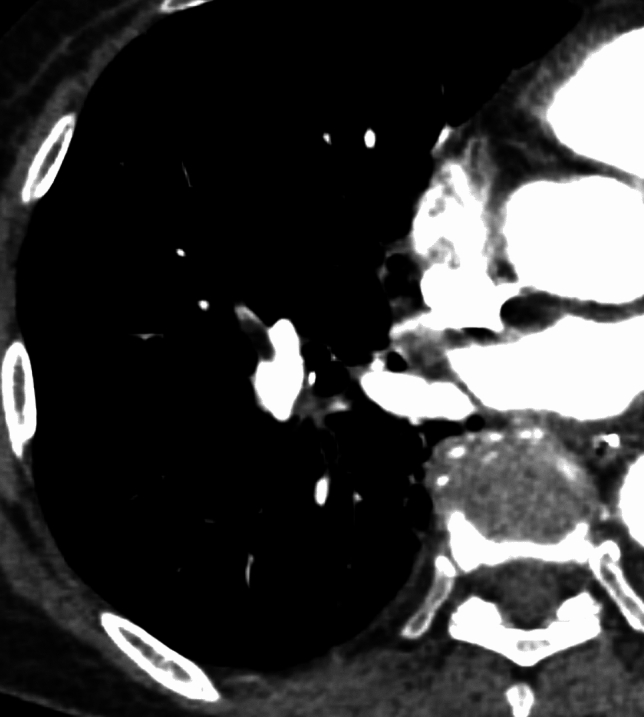
Macroscopic pulmonary fat embolism (FE). The patient was an 80-year-old woman who experienced cardiac arrest during the prosthetic replacement of the femoral stem. Contrast-enhanced CT after the return of spontaneous circulation showed multiple filling defects with a negative CT value (− 86 HU) in the pulmonary arteries

Small centrilobular nodules and the ‘crazy-paving’ pattern may also be observed in FE cases [4]. These nodules are less common but typically occur in the peripheral and upper lobes of a lung, often distributed in the subpleural regions and along the interlobular septa. They usually appear early in an FE, reflecting neovascularization following mechanical obstruction, and they subsequently resolve [12]. Fat-density filling defects are occasionally seen in the pulmonary arteries; however, large FE can be fatal and are rarely detected on antemortem CT scans [7, 11]. Figure 3 illustrates such a case.

### FE: differential diagnoses and pitfalls

The differential diagnoses for FE include ARDS, pulmonary contusion, aspiration pneumonia, and diffuse alveolar hemorrhage [1, 4]. Distinguishing an FE from ARDS can be challenging, as their imaging findings overlap. However, an FE often presents earlier and may show smaller, more peripheral opacities [1]. To differentiate FE and lung contusion, the timing of the radiographic findings after the trauma can be helpful. Lung contusions are usually visible immediately, while an FE tends to appear 24–48 h after trauma [10, 19]. Importantly, the absence of overt trauma does not exclude the possible presence of an FE, particularly in cases related to pancreatitis or medical procedures [22, 23].

## Tumor embolism and pulmonary tumor thrombotic microangiopathy

### Overview and pathogenesis

A pulmonary tumor embolism (PTE) involves the occlusion of pulmonary arteries by malignant cells, most commonly originating from extrapulmonary cancers such as breast, gastric, renal cell, or liver cancers, choriocarcinomas, and sarcomas [7]. PTEs are classified into (*i*) macroemboli, in which tumor cells occlude larger arteries, and (*ii*) microemboli, which involve smaller vessels and include pulmonary tumor thrombotic microangiopathy (PTTM). PTTM is characterized by tumor emboli leading to fibrocellular intimal proliferation, microvascular thrombosis, and pulmonary arterial hypertension (PAH) [24, 25]. PTTM is most commonly associated with mucin-producing adenocarcinomas, especially gastric cancer, but it also occurs in breast, lung, and urothelial carcinomas [1, 24]. The pathogenesis of PTTM involves tumor-derived procoagulant and angiogenic factors promoting endothelial injury, thrombosis, and progressive pulmonary hypertension [24, 25].

### PTE and PTTM: image findings

**Fig. 4:**
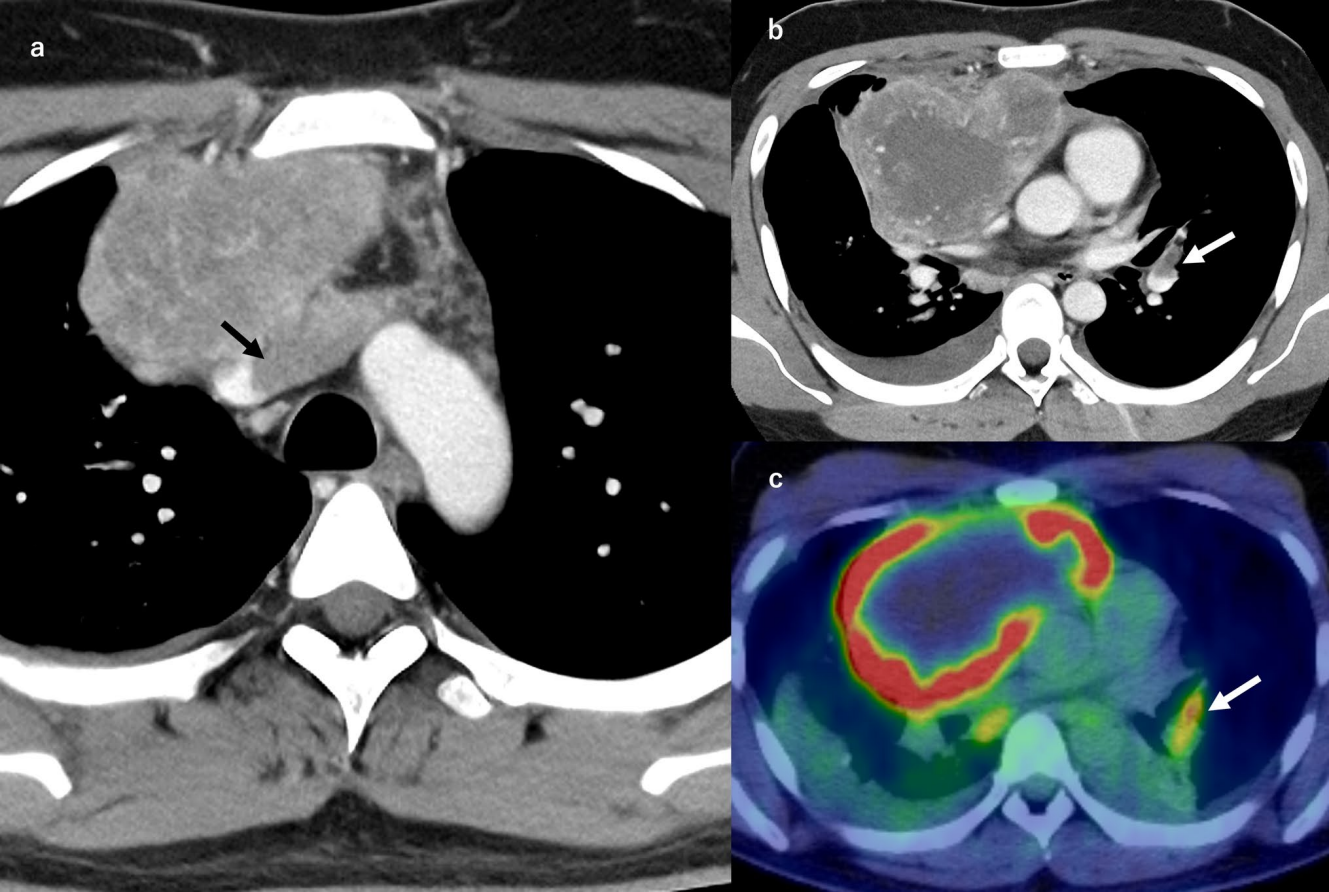
Macroscopic pulmonary tumor embolism (PTE). A man in his 30 s was diagnosed with a macroscopic PTE originating from an anterior mediastinal germ cell tumor. Contrast-enhanced CT revealed a large anterior mediastinal mass with heterogeneous internal enhancement (**a**, **b**) and significant FDG uptake (SUVmax 19.72) (**c**). The mass was seen infiltrating the left brachiocephalic vein (**a**). A tumor embolus with faint contrast enhancement was also noted in the left pulmonary artery (**b**). This intravascular lesion also demonstrated FDG accumulation (SUVmax 5.66), consistent with a tumor embolism (**c**)

**Fig. 5:**
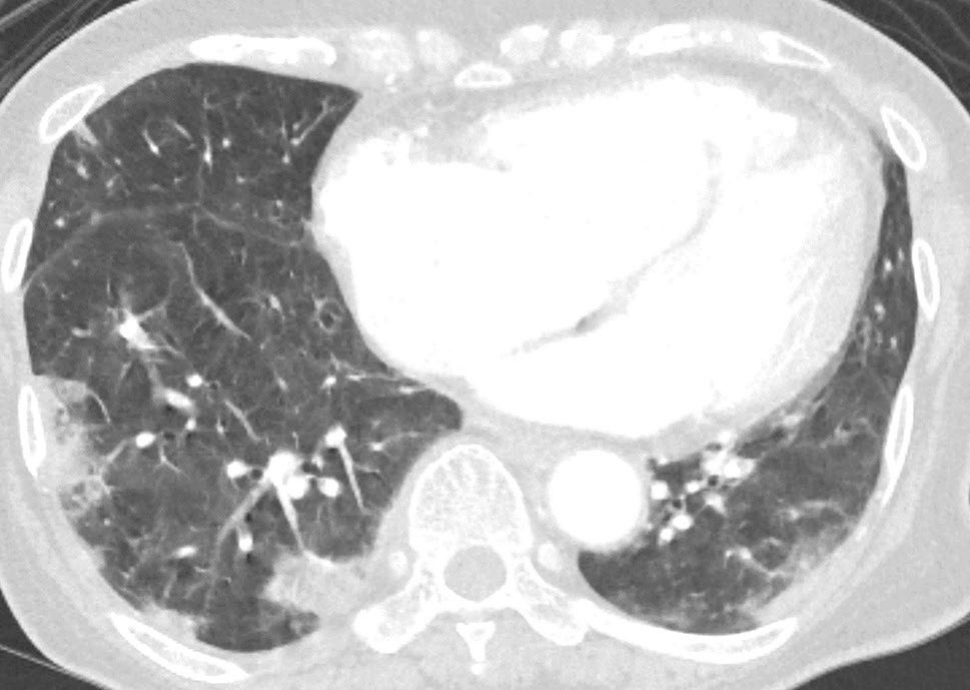
Microscopic pulmonary tumor embolism. A woman in her 60 s was diagnosed with a microscopic pulmonary artery tumor embolism secondary to cervical cancer. Computed tomography pulmonary angiography revealed no obvious filling defects in the pulmonary arteries, but right ventricular dilation and leftward deviation of the interventricular septum were observed. Multiple peripheral wedge-shaped nodules reflecting pulmonary infarction were identified in both lungs

The imaging features of PTE depend on the size and distribution of the emboli. CTPA may reveal filling defects in central arteries mimicking a thromboembolism, especially in cases of a macroembolism, as shown in the case in Fig. 4 [19]. The following findings may be observed in a microembolism case: multifocal pulmonary infarction, tree-in-bud opacities, and small centrilobular nodules (Fig. 5) [8, 19, 26]. Computed tomography may also reveal associated radiologic findings of malignancy, including lymphadenopathy, pulmonary venous hypertension, and lymphangitic carcinomatosis [16].

**Fig. 6:**
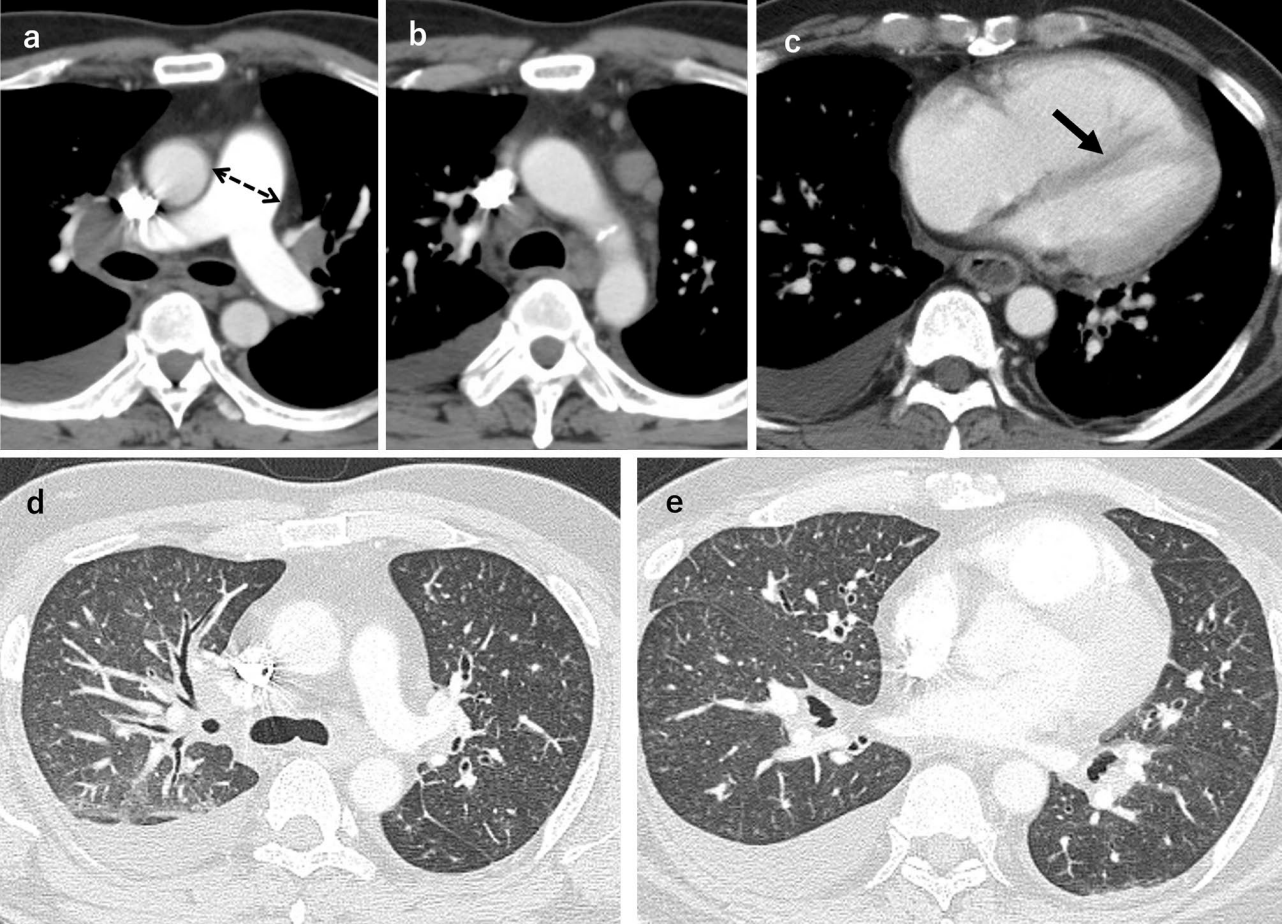
Pulmonary tumor thrombotic microangiopathy (PTTM). A man in his 40 s was diagnosed with PTTM secondary to a small gastric carcinoma at autopsy. Contrast-enhanced CT revealed pulmonary artery dilatation (**a**), mediastinal lymphadenopathy (**b**), right ventricular enlargement, leftward deviation of the interventricular septum and pleural effusion (**c**). The pulmonary findings included small centrilobular nodules, interlobular septal thickening, and thickening of the bronchovascular bundles (**d**–**e**)

PTTM typically demonstrates nonspecific GGOs, septal thickening, small nodules, and signs of pulmonary hypertension, as revealed in Fig. 6 [25]. PTTM should be suspected in the cases of cancer patients with hypoxemia but few abnormal findings in the lung fields, and signs of pulmonary hypertension (e.g., right heart enlargement) should be evaluated. V/Q scans or dual-energy CT-derived iodine perfusion maps can identify subpleural perfusion defects correlating with small-vessel occlusion, enhancing diagnostic confidence [2, 25, 27]. Fluorodeoxyglucose-positron emission tomography/computed tomography (FDG-PET/CT) may reveal hypermetabolic activity in embolic lesions but has limited specificity due to the possible overlap with inflammatory processes [8, 25].

### PTE and PTTM: differential diagnoses and pitfalls

The primary differential diagnoses include PTE, chronic thromboembolic pulmonary hypertension (CTEPH), intravascular malignant lymphoma, lymphangitic carcinomatosis, and infectious bronchiolitis, particularly when tree-in-bud patterns are present [8, 19, 28, 29]. Differentiating tumor emboli from thrombi is challenging, as both may appear as hypodense filling defects on CT. The estimated frequency of incidental pulmonary embolism among patients with cancer is 3.4%, and tumor emboli is a less common condition compared to PTE [30]. Features favoring tumor embolism include persistent filling defects despite anticoagulation and enhancing intravascular soft tissue on delayed imaging [7, 19]. Awareness of these imaging features is essential, as early recognition may allow initiation of treatment, including chemotherapy or corticosteroids, which may improve outcomes in selected PTTM [25].

## Septic embolism

### Overview and pathogenesis

**Fig. 7:**
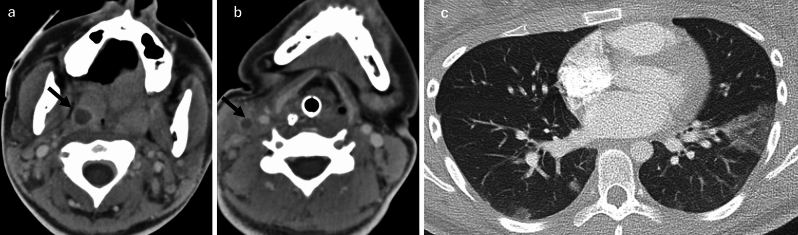
Septic embolism with Lemierre’s syndrome. A woman in her 20 s was diagnosed with a septic embolism secondary to Lemierre’s syndrome. Contrast-enhanced CT of the neck revealed an abscess in the right palatine tonsil (**a**) and thrombosis of the right internal jugular vein (**b**). Chest CT demonstrated a triangular, wedge-shaped ground-glass opacity with well-defined margins abutting the pleura in the left lower lobe; these findings are consistent with pulmonary infarction. The periphery of the opacity was denser than the center. Multiple peripheral nodules were also noted in the right lower lobe (**c**)

A septic pulmonary embolism (SPE) is characterized by the embolization of infected thrombi containing pathogens into the pulmonary arterial circulation, resulting in pulmonary infarctions or focal abscesses. SPEs have often arisen secondary to the presence of an indwelling intravascular device, infective endocarditis (especially tricuspid endocarditis), and soft tissue infections [31]. The causative pathogens commonly include *Staphylococcus aureus* (both methicillin-sensitive *S. aureus* [MSSA] and methicillin-resistant *S. aureus* [MRSA]), *Candida* species, and Gram-negative bacteria such as *Klebsiella pneumoniae*, especially in liver abscess cases [31]. *Fusobacterium* is frequently implicated in Lemierre's syndrome (Fig. 7), which is an anaerobic thrombophlebitis of the internal jugular vein accompanied by metastatic infection [1, 31]. Patients with hematological neoplasia are at high risk of developing an infective SPE due to fungal emboli caused by *Aspergillus*, *Mucor*, or *Candida* [1, 3].

### SPE: image findings

**Fig. 8:**
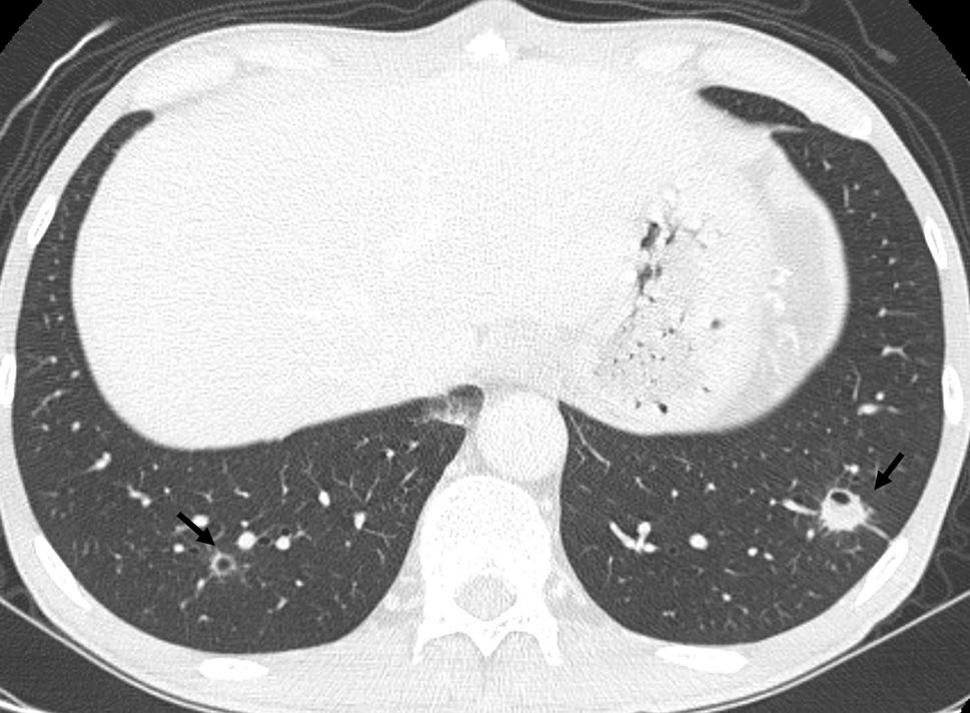
Septic embolism due to methicillin-sensitive Staphylococcus aureus (MSSA). A man in his 40 s developed a septic embolism secondary to a tonsillar abscess caused by MSSA. Chest CT revealed cavitary nodules in the peripheral regions of both lower lung lobes. A cavity in the right lower lobe shows an air-fluid level

Chest CT is the modality of choice for imaging SPEs, and it typically reveals bilateral peripheral nodules, frequently with cavitation (Fig. 8), reflecting septic infarction and abscess formation [31]. Other SPE findings include focal or wedge-shaped infiltrates, feeding vessel signs, and pleural effusions, sometimes complicated by empyema [31]. Nodules are typically seen in subpleural and gravity-dependent regions, and cavitation occurs in as many as 56% of SPE cases [2, 31]. The feeding vessel sign, although not pathognomonic, supports the diagnosis when present. The CT halo sign, i.e., a central pulmonary nodule surrounded by GGO secondary to adjacent parenchymal hemorrhage or infarction, is another nonspecific feature of SPE [1, 10, 19]. A vegetation may occasionally be present on the tricuspid valve, or an infectious source may be indicated by a thrombosed vein or a catheter-related thrombus [6, 32].

### SPE: differential diagnoses and pitfalls

The primary imaging-based differential diagnoses for SPE include thrombotic pulmonary embolism, which lacks systemic signs of infection and typically does not present with cavitation. Other differential diagnoses include cavitary pulmonary metastasis, rheumatoid lung nodules, bacterial pneumonia, granulomatosis with polyangiitis, and lymphomatoid granulomatosis [7, 31, 32]. The diagnostic pitfalls include attributing nodular infiltrates solely to malignancy or a thrombotic embolism without considering the possibility of infectious emboli, especially in high-risk populations (e.g., patients with catheters or known infections). Blood cultures, echocardiography, and a thorough assessment of potential infectious foci are essential for the accurate diagnosis of an SPE [31].

## Parasitic embolism

### Overview and pathogenesis

The term ‘parasitic embolism’ refers to vascular obstruction caused by parasitic organisms or their components. *Dirofilaria immitis* (heartworm), a filarial nematode that affects primarily dogs, can incidentally infect humans through mosquito bites. In humans, immature worms can embolize to the pulmonary arteries, where they elicit granulomatous inflammation forming necrotic nodules [33].

Echinococcosis (a hydatid disease) is a parasitic infection resulting from exposure to small tapeworms. It is categorized into two main types: cystic echinococcosis (due to *Echinococcus granulosus*) and alveolar echinococcosis (*E. multilocularis*). Humans acquire echinococcosis through the oral intake of parasite eggs shed in the feces of definitive host animals. After hatching in the intestine, the larval forms penetrate the intestinal wall and reach the liver through the portal venous system, where they form lesions [34]. A PE results from a rupture or the migration of *Echinococcus* into the pulmonary arterial circulation, often secondary to a hepatic lesion or cardiac involvement [4, 5, 35–37]. Pulmonary vascular disease associated with the parasitic nematode *Schistosoma mansoni* differs mechanistically: egg embolization via portosystemic shunts or chronic immune-mediated vasculopathy causes pulmonary hypertension [16, 38]. Unlike the mechanical obstruction seen in dirofilariasis or echinococcosis, schistosomiasis-associated PAH involves vascular remodeling driven by Th2 cytokines and transforming growth factor-beta (TGF-β) signaling [38].

### Parasitic embolism: image findings

**Fig. 9:**
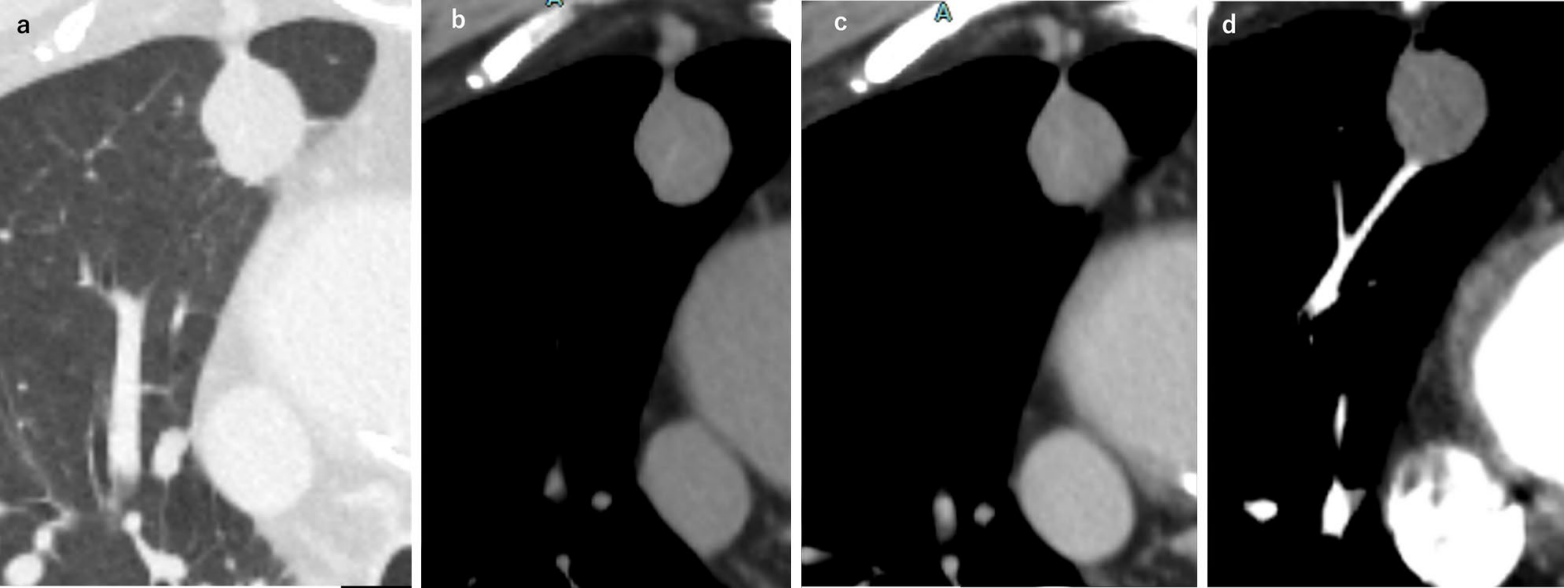
Parasitic embolism—pulmonary dirofilariasis. A man in his 70 s was diagnosed with pulmonary dirofilariasis. A solid nodule with well-defined margins was identified in the peripheral region of the right upper lobe (**a**). Non-contrast CT demonstrated faint internal calcification (**b**), and contrast-enhanced imaging showed no enhancement (**c**). The pulmonary artery adjacent to the lesion was continuous (**d**)

**Fig. 10:**
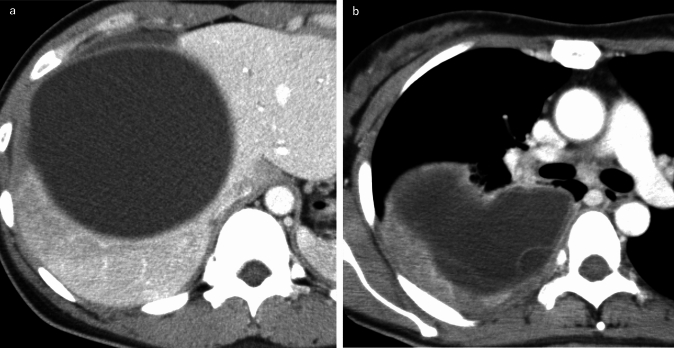
Parasitic embolism—cystic echinococcosis. A woman in her 30 s with a history of residing in the UK presented a year earlier with cough and right-sided chest and abdominal pain. Contrast-enhanced CT revealed a large cystic mass in the patient’s liver (**a**) and another cystic lesion in the right lower lobe of the lung (**b**), containing internal septations. Serologic testing confirmed cystic echinococcosis

As illustrated in Fig. 9, in cases of pulmonary dirofilariasis, CT typically reveals solitary, well-circumscribed, peripheral nodules predominantly in the right lower lobe of the lung [33, 39]. The lesions measure 8–25 mm and may mimic neoplastic nodules. Histologically, necrotic nodules with granulomatous inflammation and embedded degenerated worms are characteristic findings [33]. A cystic echinococcosis embolism manifests on CT as multiple intra-arterial cystic filling defects or peripheral nodules (Fig. 10). Cyst rupture may result in complex cavitary lesions or air-crescent signs [4, 5].

In alveolar echinococcosis, pulmonary lesions typically manifest as low-attenuation masses with lobulated or irregular margins. Intralesional and/or peripheral calcifications may be observed. These lesions can be solitary or multiple, occur unilaterally or bilaterally, and demonstrate variable size [37]. In schistosomiasis-associated PAH, chest CT may show enlarged pulmonary arteries, mosaic perfusion, and right ventricular hypertrophy; usually, no direct visualization of eggs or emboli is possible. Magnetic resonance imaging (MRI) and echocardiography are useful in assessments of right ventricular dysfunction [38].

### Parasitic embolism: differential diagnoses and pitfalls

Dirofilariasis nodules can be misinterpreted as primary lung cancer or metastatic lesions, especially in asymptomatic patients with incidental findings. In many cases, the absence of eosinophilia may mislead clinicians [33]. Cystic pulmonary echinococcosis must be differentiated from thromboembolism, tumor emboli, and septic emboli. Alveolar pulmonary echinococcosis can mimic pulmonary metastases and granulomatous diseases affecting the lungs [37]The presence of cystic lesions elsewhere supports the diagnosis, as does a prior history of a hepatic hydatid disease [5]. Schistosomiasis-associated PAH can mimic idiopathic or connective tissue disease-associated PAH. The absence of detectable eggs in modern autopsy cases emphasizes the importance of obtaining an epidemiologic history and serology findings.

The imaging findings for parasitic embolism overlap significantly with other causes of pulmonary hypertension [38]. Key diagnostic pitfalls for parasitic embolism include under-recognition of parasitic diseases in non-endemic regions and the misinterpretation of chronic granulomatous lesions as malignancy.

## Iatrogenic embolism

### Overview and pathogenesis

**Fig. 11:**
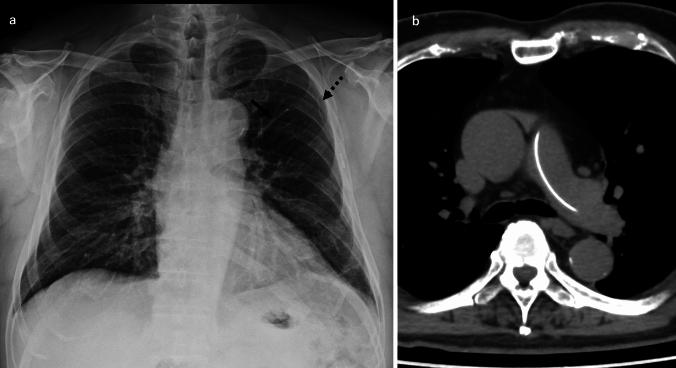
Catheter embolism. A man in his 60 s had had a port catheter placed in his left subclavian vein for chemotherapy. Chest radiography confirmed the port reservoir in the patient's left anterior chest, but the catheter appeared to have fractured (**a**, dot allow). The distal fragment had migrated into the upper lobe branch of the left pulmonary artery, with its tip located in the upper left lung field (**a**, arrow). Contrast-enhanced CT revealed a linear, hyperdense foreign body within the left pulmonary artery, consistent with the fractured catheter tip (**b**)

**Fig. 12:**
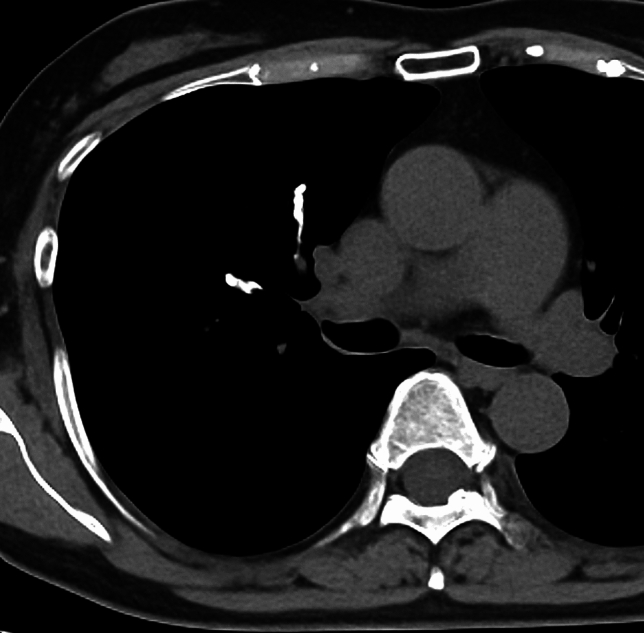
N-butyl-2-cyanoacrylate (NCBA) pulmonary embolism. A woman in her 60 s underwent the embolization of a renal arteriovenous fistula with the use of NBCA-Lipiodol®. During the embolization procedure, embolic material migrated from the dilated renal vein into the inferior vena cava. Chest CT subsequently revealed a hyperdense foreign body in the right pulmonary artery, consistent with a pulmonary embolism caused by NBCA-Lipiodol migration

An iatrogenic embolism develops when iatrogenic materials migrate into the pulmonary circulation. Common sources include fragments of intravascular devices such as catheters (Fig. 11), guidewires, vena cava filters, embolization coils, and brachytherapy seeds [2, 9, 10]. Pathophysiologically, these foreign materials can cause purely mechanical obstruction, trigger local thrombosis, and (rarely) provoke inflammatory responses [5, 9]. Embolic materials such as polymethymethacrylate (cement) for vertebroplasty, N-butyl-2-cyanoacrylate (NBCA) as described for the case in Fig. 12, and iodinated oil for thoracic duct embolization or hepatocellular carcinoma chemoembolization can also cause an iatrogenic pulmonary artery embolism [4, 5, 11]. Migration mechanisms include fragmentation during catheter manipulation, the pinch-off syndrome, and migration through venous plexuses in the case of seeds or cement [5].

### Iatrogenic emboli: image findings

Radiography and CT are the primary modalities for the detection of iatrogenic emboli. CTPA is the gold standard for their localization and characterization. Most intravascular foreign bodies appear as high-density, hyperattenuating linear or punctate structures within the pulmonary arteries or right heart chambers [2, 9, 10]. Computed tomography bone windows enhance the visualization of metallic objects, e.g., guidewires, coils, and brachytherapy seeds [9]. Cement and NBCA emboli present as tubular or branching hyperdense opacities in the pulmonary arteries [2, 7]. Advanced CT techniques including multiplanar reconstruction (MPR) and maximum intensity projection (MIP) are invaluable for identifying small or complex foreign bodies [9].

### Iatrogenic emboli: differential diagnoses and pitfalls

**Fig. 13:**
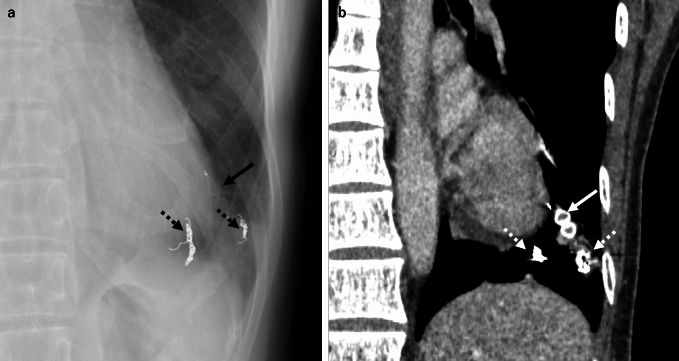
Vascular plug and coil placed in the pulmonary artery to treat a pulmonary arteriovenous fistula. A woman in her 40 s underwent the embolization of a pulmonary arteriovenous fistula in the left lower lobe of her lung with the use of a vascular plug and coils. Chest radiography and chest CT confirmed the presence of the vascular plug (**a**, **b**, arrow) and coils within the left pulmonary artery (**a**, **b**, dot arrow)

The differential diagnoses for an iatrogenic embolus include thrombotic embolus, which usually lacks hyperattenuating components, and a calcified embolus from a chronic infection or metastatic disease. A calcified fibrin sheath, which is a complication of the long-term use of a central venous catheter, may mimic an embolized foreign body [9]. Radiologists must differentiate true emboli from normally deployed intravascular devices, such as ambulatory pulmonary artery pressure-monitoring devices (e.g., the CardioMEMS™) and vascular plugs (Fig. 13) [9, 10]. Artifacts from metal streaking can obscure small foreign bodies, underscoring the importance of optimal windowing and the use of prior imaging for comparison. The misinterpretation of non-radiopaque materials or overlying devices as emboli also poses diagnostic challenges [9].

## Gas embolism

### Overview and pathogenesis

**Fig. 14:**
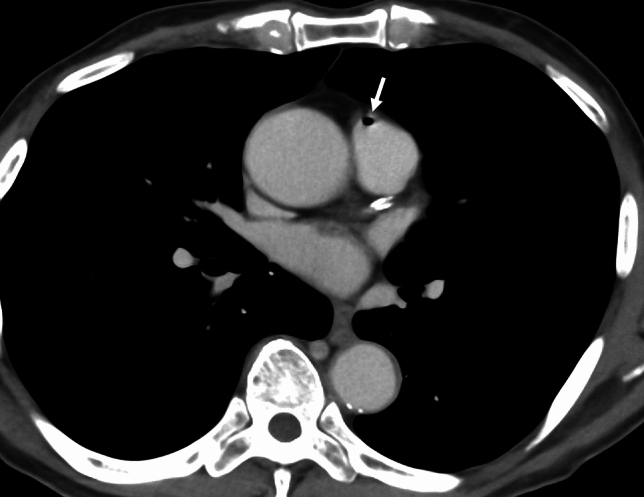
Air embolism after undergoing contrast-enhanced CT. An 80-year-old male underwent an examination by contrast-enhanced CT, which revealed a filling defect with a negative CT value (− 730 HU) in the main pulmonary artery. The amount of air was small and not clinically significant

An air or gas embolism arises when gas enters the vasculature, obstructing blood flow and potentially causing ischemia. These emboli are categorized as venous air emboli and arterial air emboli. A venous air embolism commonly occurs during invasive procedures, including central venous catheter insertion, trauma, and surgical interventions [40, 41]. An air in the vessels has been observed in up to 23% of patients undergoing a contrast-enhanced CT examination, though such emboli are usually minimal and clinically insignificant (Fig. 14) [2]. An arterial air embolism can result from a paradoxical embolism through right-to-left shunts (e.g., patent foramen ovale) or directly into the artery [7, 40].

A gas embolism sometimes occurs in deep-sea divers; this has been referred to as ‘decompression illness’ [3, 4, 42]. In decompression illness, a rapid reduction in the ambient pressure leads to the formation of nitrogen bubbles within the vasculature and tissues, causing vascular obstruction, endothelial damage, and systemic inflammatory responses [3, 42].

### Air embolism: image findings

**Fig. 15:**
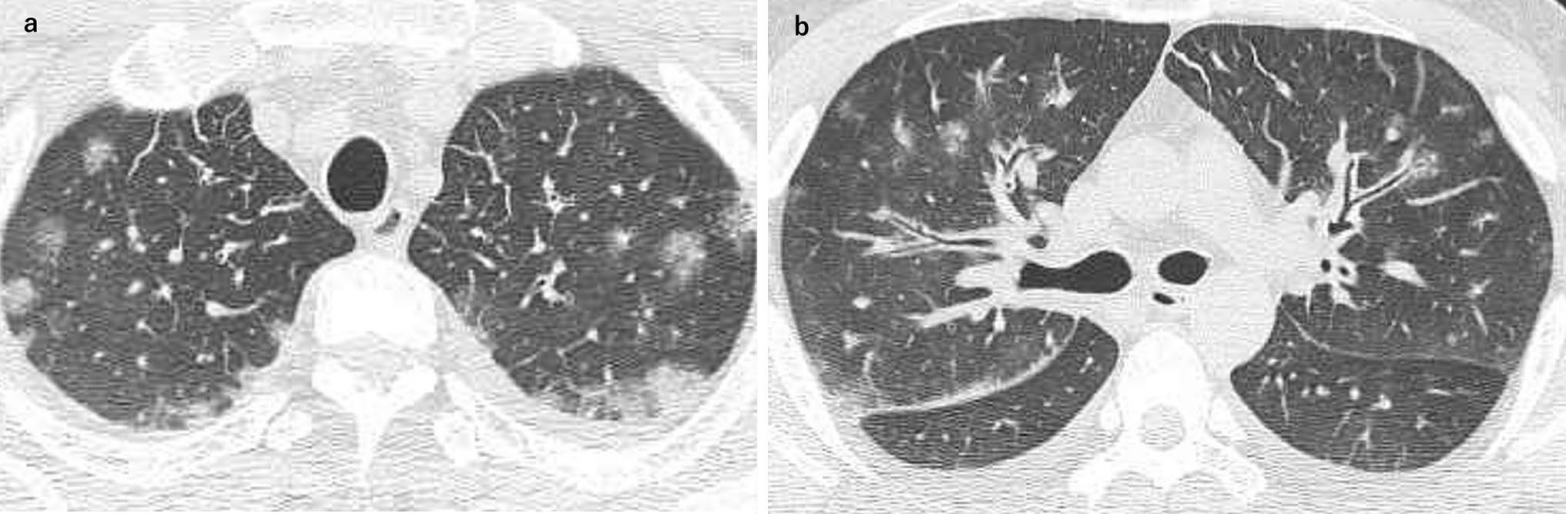
Decompression illness. A male fisherman in his 30 s surfaced after working at a depth of 35 m. At 30 min after he came to the surface, he experienced numbness in his hands and feet, followed by respiratory symptoms. He was diagnosed with decompression illness. Chest CT revealed multiple peripheral ground-glass nodules in both lungs, along with interlobular septal thickening (**a**) and thickening of the bronchovascular bundles (**b**)

On CT, intravascular gas appears as low-attenuation foci in the right atrium, right ventricle, pulmonary arteries, and/or systemic circulation. A paradoxical air embolism may manifest as air within the systemic arteries, sometimes without direct cardiac defects [40]. In individuals with decompression illness, CT may reveal gas bubbles in the pulmonary vasculature, cerebral vessels, and/or systemic veins, particularly in severe cases. However, CT has often been found to be unable to show any evidence of intravascular gas in patients with decompression illness [1]. Additional findings may include pulmonary hypertension, right ventricular strain, and/or pulmonary edema (Fig. 15) [5].

### Air embolism: differential diagnoses and pitfalls

The differential diagnoses for an air embolism include PTE and FE. Small volumes of iatrogenic air, especially after invasive medical procedures, may be clinically insignificant but should be differentiated from a pathologic embolism. A critical pitfall is the potential disappearance of gas bubbles on delayed imaging, which can lead to missed diagnoses [40]. In decompression illness, mild cases may lack radiologic findings despite the presence of significant clinical symptoms. Early imaging and correlation with the clinical context are essential for the accurate diagnosis of an air embolism.

## Conclusion

The group termed ‘nonthrombotic pulmonary embolism (NTPE)’ includes diverse entities: fat embolism, tumor embolism, and pulmonary tumor thrombotic microangiopathy, air embolism, amniotic fluid embolism, septic embolism, parasitic embolism, and foreign body embolism. Despite their varied etiologies, NTPEs pose diagnostic challenges and can result in significant morbidity and mortality. Imaging, particularly by CT, is essential for detecting vascular and parenchymal abnormalities. Radiologists must recognize imaging features such as right heart strain and mosaic perfusion, and it is necessary to differentiate an NTPE from a thrombotic embolism and various differential diagnoses. The early diagnosis of an NTPE facilitates its appropriate treatment and may improve outcomes.
